# Relationship between Erythrocyte Omega-3 Content and Obesity Is Gender Dependent

**DOI:** 10.3390/nu6051850

**Published:** 2014-05-05

**Authors:** Peter R. C. Howe, Jonathan D. Buckley, Karen J. Murphy, Tahna Pettman, Catherine Milte, Alison M. Coates

**Affiliations:** 1 Nutritional Physiology Research Centre, University of South Australia, GPO Box 2471, Adelaide, SA 5001, Australia; E-Mails: Jon.Buckley@unisa.edu.au (J.D.B.); karen.murphy@unisa.edu.au (K.J.M.); tpettman@unimelb.edu.au (T.P.); catherine.milte@deakin.edu.au (C.M.); alison.coates@unisa.edu.au (A.M.C.); 2 Clinical Nutrition Research Centre, University of Newcastle, Callaghan, NSW 2308, Australia

**Keywords:** Omega-3 Index, adiposity, fish oil, gender

## Abstract

Epidemiological evidence of an inverse association between consumption of long-chain omega-3 polyunsaturated fatty acids (LC *n*-3 PUFA) and obesity has been conflicting, even though studies in animal models of obesity and limited human trials suggest that LC *n*-3 PUFA consumption may contribute to weight loss. We used baseline data from a convenience sample of 476 adults (291 women, 185 men) participating in clinical trials at our Centre to explore relationships between erythrocyte levels of LC *n*-3 PUFA (a reliable indicator of habitual intake) and measures of adiposity, viz. body mass index (BMI), waist circumference (WC) and body fat (BF) assessed by dual-energy X-ray absorptiometry. Means ± SD of assessments were BMI: 34 ± 7 and 31 ± 5 kg/m^2^; WC: 105 ± 16 and 110 ± 13 cm; BF: 48 ± 5 and 35% ± 6% in women and men respectively. Erythrocyte levels of eicosapentaenoic acid (EPA) and docosahexaenoic acid (DHA) were similar in men and women while docosapentaenoic acid (DPA) was higher and EPA + DHA (Omega-3 Index) slightly lower in men than in women. Both DHA and EPA + DHA correlated inversely with BMI, WC and BF in women while DPA correlated inversely with BF in men. Quartile distributions and curvilinear regression of the Omega-3 Index *versus* BMI revealed a steep rise of BMI in the lower range of the Omega-3 Index in women, but no association in men. Thus the results highlight important gender differences in relationships of specific LC *n*-3 PUFA in erythrocytes to markers of adiposity. If these reflect causal relationships between LC *n*-3 PUFA consumption and risk of obesity, gender specific targeted interventions should be considered.

## 1. Introduction

Polyunsaturated fatty acids (PUFA) are known to beneficially influence fat metabolism and there are numerous studies in animal models of obesity showing that consumption of PUFA, particularly the long-chain omega-3 (LC *n*-3) PUFA from marine sources, can increase fat loss and counteract adiposity [1,2]. This has been supported by a limited number of human trials of LC *n*-3 PUFA supplementation [1,2,3], although epidemiological evidence of an inverse association between consumption of LC *n*-3 PUFA and obesity has been conflicting [4,5].

In the Health Professionals Follow-Up Study, men with high fish consumption were less likely to be overweight than those with low fish consumption and the proportion of overweight volunteers was inversely related to LC *n*-3 PUFA intake [4]. The Nurses’ Health Study, on the other hand, found that higher intakes of fish and LC*n*-3 PUFA were associated with a higher prevalence of obesity [5]. While this unexpected effect of fish intake could be accounted for by higher energy intakes, this was not the case for LC *n*-3 PUFA intake. However, both these large studies estimated dietary intakes from semi-quantitative food frequency questionnaires which are limited in their ability to accurately assess intakes of different types of fat, particularly LC *n*-3 PUFA.

An alternative approach to assess relationships between LC *n*-3 PUFA consumption and obesity is to evaluate a surrogate biomarker of LC *n*-3 PUFA intake. Several studies have measured LC *n*-3 PUFA as a percentage of total fatty acids in plasma phospholipids with conflicting outcomes. Three early studies conducted in populations of varying ethnicity in Canada found that plasma phospholipid LC *n*-3 PUFA correlated positively with waist circumference [6,7,8] whereas more recent studies have reported inverse correlations with measures of adiposity [9,10,11].

Fatty acid levels in plasma phospholipids reflect consumption of dietary fatty acids over a relatively short period (weeks), whereas erythrocyte levels reflect intake over several months [12,13]. Hence the latter is regarded as the most reliable surrogate marker of habitual dietary intake of LC *n*-3 PUFA. Docosahexaenoic acid (DHA), in particular, is incorporated and retained predominantly inside the plasma membrane for the 4 month life of the erythrocyte [12]. Surprisingly, there is little information on relationships between erythrocyte LC *n*-3 PUFA levels and adiposity, although a recent analysis of a cohort of almost 3000 subjects from the Framingham Heart Study indicated a modest inverse relationship between erythrocyte LC *n*-3 PUFA and waist circumference [14].

As we routinely measure erythrocyte fatty acids in nutritional intervention trials, we have chosen to examine relationships between erythrocyte LC *n*-3 PUFA levels and measures of adiposity in baseline data obtained from a convenience sample of trial participants, most of whom had undergone DEXA assessments of body composition. In particular, we have sought to explore potential gender differences in such relationships.

## 2. Methods

### 2.1. Participants and Data

A secondary analysis was undertaken using de-identified pooled data obtained from volunteers who had participated in nutritional intervention trials conducted by the University of South Australia’s Nutritional Physiology Research Centre between 2005 and 2009. Five trials were selected in which measures of weight and adiposity, together with analysis of erythrocyte fatty acid levels, had been undertaken at baseline. Each trial had been approved by the University’s Human Research Ethics Committee.

Participants were free-living, non-smoking men and women from both metropolitan and regional locations who were predominantly overweight/obese (inclusion criterion for three of the five trials) but otherwise healthy (*i.e.*, without a diagnosed disease condition) and had limited consumption of fish or fish oil (inclusion criterion for four trials). Baseline anthropometric measurements and blood samples for determination of erythrocyte fatty acid profiles were obtained from 476 participants prior to undergoing dietary interventions. Additionally, dual-energy X-ray absorptiometry (DEXA) assessments of body composition were obtained at the same time from 376 of these participants.

### 2.2. Assessments

#### 2.2.1. Anthropometric Measurements

Each participant’s height and weight were recorded to calculate body mass index (BMI). Height was measured to the nearest 0·1 cm whilst barefoot using a wall-mounted stadiometer (SECA; Vogel & Halke, Hamburg, Germany). Body weight was measured to the nearest 0.1 kg with participants wearing light clothing using a TANITA Ultimate Scale 2000 (Tanita Corporation, Tokyo, Japan). Waist circumference was measured using a metric tape according ISAK international guidelines [15].

#### 2.2.2. Body Composition

Each participant underwent a whole body DEXA scan (Lunar Prodigy, General Electric, Madison, WI, USA) to determine fat mass and lean mass, from which percentage body fat was estimated.

#### 2.2.3. Assessment of Fatty Acid Profiles

Relative proportions of individual fatty acids in erythrocytes were assessed using a procedure adapted from previously published methods [16]. Erythrocytes were isolated within 2 h of collection by centrifugation, washed in isotonic saline and stored at −80 °C. They were subsequently thawed and the lipids were extracted with chloroform and isopropanol (2:1). The organic phase containing the lipid was evaporated to dryness under a stream of N_2_ gas. The lipids were then transesterified with acetyl chloride in methanol toluene (4:1, *v*/*v*) at 100 °C for 1 h. The resultant fatty acid methyl esters were extracted with 10% potassium carbonate. Fatty acid methyl esters were separated and quantified using a Shimadzu 2010 gas chromatograph equipped with a 50 m capillary column (0·32 mm, inner diameter) coated with BPX-70 (0·25 mm film thickness; SGE Analytical Science Pty Ltd., Ringwood, VIC, Australia). The injector temperature was set at 250 °C and the detector (flame ionisation) temperature at 260 °C. The initial oven temperature was 130 °C and was programmed to rise to 220 °C at 58 °C/min. H_2_ was used as the carrier gas at a velocity of 36.4 cm/s. Fatty acid methyl esters were identified based on the retention time to authentic lipid standards (GLC-463; Nu-Chek Prep, Inc., Elysian, MN, USA).

Erythrocyte contents of eicosapentaenoic acid (EPA), docosapentaenoic acid (DPA) and docosahexanoic acid (DHA) were expressed as percentages of total erythrocyte fatty acids. The Omega-3 Index was calculated as the sum of the EPA and DHA contents.

### 2.3. Statistical Analysis

Data were analysed using SPSS for Windows (Version 21.0, 2012) and presented as means ± SD (standard deviations). Gender differences in outcome measures were determined by Student’s *t*-test; statistical significance was set at *p* < 0.05. Relationships between markers of adiposity and erythrocyte fatty acid contents were assessed by correlation analysis and expressed as Pearson correlation coefficients (*r*). A Bonferroni correction was made for comparisons of each adiposity measure with multiple fatty acids whereby statistical significance was set at *p* < 0.01. Univariate models were used to test for gender interactions. Quartiles of Omega-3 Index were determined for each gender and mean BMI values for each quartile were compared by ANOVA with statistical significance set at *p* < 0.05.

## 3. Results

### 3.1. Participant Characteristics

Participants were middle-aged and predominantly women. Table 1 presents anthropometric and body compositional assessments for each gender. Due to the selection of overweight/obese adults for the majority of clinical trials, average values of BMI fell within obese classifications for men and women, although there was a wide range (18–59 kg/m^2^).

**Table 1:** Participant characteristics *.

	Males	Females
Age (year)	45.6 ± 11.6 (185)	47.5 ± 12.3 (291)
Weight (kg)	99.4 ± 17.3 (185)	91.3 ± 19.9 (291)
Height (m)	177.7 ± 7.0 (185)	163.6 ± 6.8 (291)
Body mass index (kg/m^2^)	31.4 ± 5.0 (185)	34.0 ± 6.8 (291)
Waist circumference (cm)	110.0 ± 13.3 (133)	105.3 ± 16.1 (244)
Fat mass (% of total mass)	34.5 ± 6.3 (133)	48.2 ± 5.2 (243)

* Data are presented as mean ± standard deviation (number of observations provided in brackets).

### 3.2. Erythrocyte Fatty Acids

Table 2 shows mean values of erythrocyte fatty acid levels for each gender. Men had significantly higher erythrocyte DPA while the Omega-3 Index (EPA + DHA) was significantly higher in women.

**Table 2:** Erythrocyte fatty acids (% of total; mean ± SD).

	Males (185)	Females (282)
EPA	0.85 ± 0.35	0.91 ± 0.42
DPA **	2.47 ± 0.37	2.32 ± 0.36
DHA	4.25 ± 0.95	4.42 ± 1.02
Omega-3 Index *	5.10 ± 1.18	5.33 ± 1.33

Significant gender difference: * *p* < 0.05; ** *p* < 0.0001.

Table 3 summarises the linear correlation analysis of relationships between erythrocyte fatty acids and measures of adiposity. Pearson correlation coefficients (r) are presented for all participants and for men and women separately. There were strong inverse correlations in the whole dataset between DHA, DPA and the Omega-3 Index and measures of adiposity. DHA and the Omega-3 Index were associated with BMI and waist circumference, whereas DPA predicted body fat. However, the apparent influence of the Omega-3 Index can be attributed to DHA alone as EPA was weakly associated with waist circumference only.

**Table 3:** Correlations between erythrocyte fatty acids and adiposity measures.

	Body mass index (kg/m^2^)	Waist circumference (cm)	Body Fat (%)
**N (all subjects)**	476	377	376
EPA	−0.016	−0.143 *	−0.054
DPA	−0.073	−0.116	−0.264 ***
DHA	−0.191 ***	−0.298 ***	−0.117
Omega-3 Index	−0.154 **	−0.275 ***	−0.108
**N (males only)**	185	133	133
EPA	0.087	−0.062	−0.147
DPA	−0.016	−0.201	−0.228 *
DHA	−0.077	−0.144	−0.185
Omega-3 Index	−0.037	−0.133	−0.192
**N (females only)**	291	244	243
EPA	0.080	−0.164 *	−0.172 *
DPA	−0.047	−0.125	−0.143
DHA	−0.276 **	−0.353 ***	−0.329 **
Omega-3 Index	−0.236 **	−0.322 ***	−0.306 **

Values are Pearson *r*. Significant correlations: * *p* < 0.01, ** *p* < 0.001, *** *p* < 0.0001.

Univariate analysis confirmed that there were significant gender interactions in the relationships between Omega-3 Index and BMI (*p* = 0.015) and Omega-3 Index and waist circumference (*p* = 0.028) but not between Omega-3 index and % body fat. Hence it was appropriate to split the data by gender, although gender differences in correlations with % body fat should be interpreted with caution. Erythrocyte DPA was the only significant correlate of adiposity in men; higher DPA predicted a lower percentage body fat. However, DPA was not a significant predictor in women. On the other hand, there were strong inverse correlations between all three markers of adiposity and DHA and consequently the Omega-3 Index in women.

### 3.3. Associations between the Omega-3 Index and BMI

**Figure 1:**
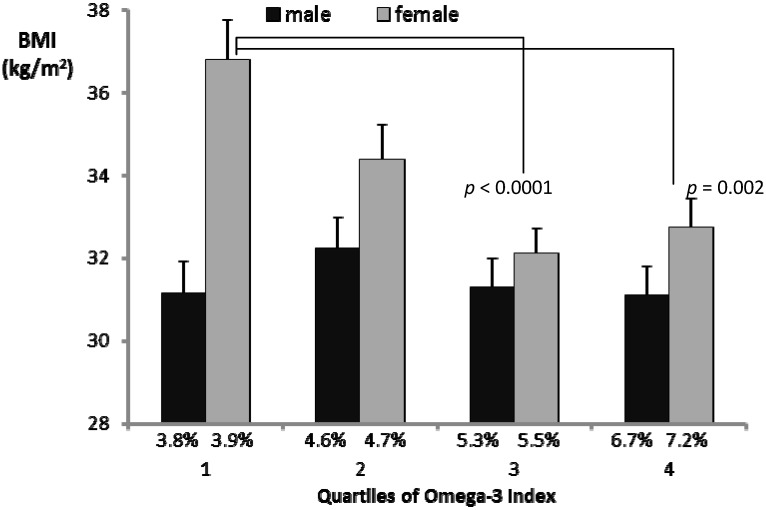
Average BMI values in quartiles of the Omega-3 Index.

BMI is the most widely used measure of obesity and the Omega-3 Index is the most widely accepted marker of habitual intake of LC *n*-3 PUFA. Hence it was of interest to further illustrate the relationship between these measures. Figure 1 shows the mean values for BMI in each quartile of the Omega-3 Index for each gender. The lack of a significant relationship in men was apparent. It was also apparent that the relationship between the Omega-3 Index and BMI in women was not linear. BMI values appeared to rise steeply in the lower quartiles of the Omega-3 Index. There were highly significant differences between mean BMI in the lowest quartile and mean BMI in the two highest quartiles of the Omega-3 Index in women.

**Figure 2:**
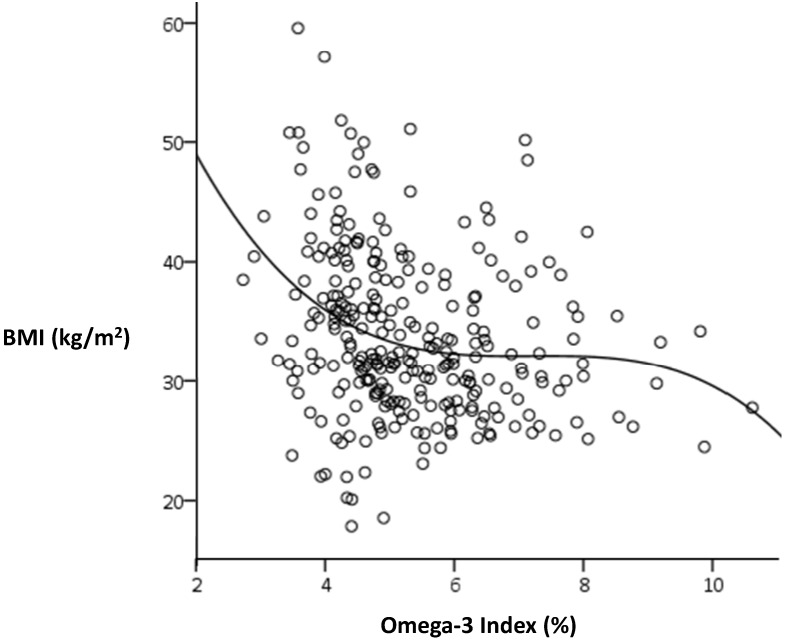
Sigmoidal relationship between BMI and the Omega-3 Index in women.

Curvilinear analysis confirmed the skewness of the relationship between Omega-3 Index and BMI in women (Figure 2). A sigmoidal curve gave the most significant fit (*r*^2^ = 0.078, *p* < 0.001) and suggested a possible threshold for the Omega-3 Index around 6%, below which BMI tends to rise steeply. There was no such relationship for men (*r*^2^ = 0.006, *p* = 0.74).

## 4. Discussion

The results of this study confirm previous indications of an inverse relationship between LC *n*-3 PUFA levels in erythrocytes and adiposity in humans [14]. Moreover, they extend previous research by revealing a primary role for DHA in this relationship. Most importantly, however, they highlight a striking gender difference, whereby the association of DHA with lower adiposity was evident in women only; men, on the other hand, tended to show an inverse association between erythrocyte DPA and adiposity. It was also apparent that erythrocyte EPA had little relationship with adiposity.

Recognising that erythrocyte levels reflect habitual intakes of LC *n*-3 PUFA, it is tempting to speculate that increased consumption of DHA-rich fish or fish oil may help to counteract obesity. However, the correlations derived from our cross-sectional analysis cannot imply causation. One could equally argue that being obese inclines individuals to include less fish or fish oil in their diet. Alternatively, lower intakes of fish or fish oil and a predisposition to adiposity may both be secondary to another independent factor, e.g., poor quality diet. The influence of independent factors may also account for previous anomalies in reported relationships of LC *n*-3 PUFA intake with adiposity. For example, the observation that larger waist circumferences were associated with higher plasma phospholipid LC *n*-3 PUFA levels in Canadian Inuits and Cree Indians may have been attributable to other aspects of diet in these populations, notwithstanding their habitually high intakes of LC *n*-3 PUFA [6,7,8].

However, preclinical research in animal models of obesity and limited data from human intervention trials suggests that LC *n*-3 PUFA consumption is causally related to adiposity. LC *n*-3 PUFA can suppress fat synthesis and increase metabolism in adipose tissue via multiple mechanisms involving altered expression of transcription factors, viz. SREBP-1 and PPARs [17]. *In-vitro* studies with lipid droplets specifically implicate DHA in these mechanisms [18]. Coincidentally, DHA was the predominant LC *n*-3 PUFA consumed in a small number of human intervention trials which reported weight loss or fat loss following supplementation [3,19,20]. Hence the highly significant inverse correlations between erythrocyte DHA and diverse measures of adiposity observed in the present study suggest that increasing DHA intake may help to reduce the incidence of adiposity (*r* = 0.353 indicates that erythrocyte DHA levels account for 12.5% of the variance of waist circumference in women).

The limitation of this association to women is noteworthy, particularly considering that there was no significant difference between men and women in the mean erythrocyte DHA level. Interestingly, Decsi and Kennedy [21] reported that plasma phospholipid DHA levels for almost 3000 participants in the EPIC study were approximately 10% higher in women than men, an effect that they attributed to enhanced conversion of α-linolenic acid through to DHA in women. However, no differences were reported for DPA. The 6% higher erythrocyte DPA level for men in the present study appears to be a unique observation but would be consistent with the hypothesis of limited conversion of DPA to DHA in men relative to women. It is of interest, therefore, that DPA was a significant predictor of body fat in men, whereas DHA was the predominant predictor of all measures of adiposity in women. Garg and colleagues recently reported that DHA supplementation was effective in reducing platelet aggregation in women, whereas EPA supplementation reduced platelet aggregation in men [22]. Clearly there is a need to further characterise gender differences in LC *n*-3 PUFA and their respective functions. A useful starting point would be large epidemiological studies such as EPIC and the Framingham Heart Study, where blood samples have been routinely analysed for LC *n*-3 PUFA contents.

There is increasing recognition of the limitations of dietary intake assessment tools to estimate LC *n*-3 PUFA intake and increasing acknowledgement of the need for reliable blood biomarkers of an individual’s LC *n*-3 PUFA status. Unfortunately the use of different biomarkers can lead to different interpretations. Thus the relatively simple measure of LC *n*-3 PUFA in whole plasma is at best a reflection of recent consumption, whereas assessment in a plasma phospholipid fraction reflects both consumption and incorporation of LC *n*-3 PUFA in a stable pool over a period of weeks. However, the “gold standard” biomarker for habitual LC *n*-3 PUFA consumption is their relative content in erythrocytes, reflecting, as stated earlier, their uptake and retention in the erythrocyte pool over several months [12,13]. It is unfortunate that a number of important epidemiological studies have chosen to use plasma phospholipid determinations when there is increasing recognition of the superiority of erythrocyte fatty acid determinations. Indeed the Omega-3 Index, *i.e.*, the sum of EPA and DHA in erythrocytes, has been widely promoted as both a biomarker of LC *n*-3 PUFA consumption/status and a risk factor for cardiovascular disease [23] and serves as a useful standard for comparison across populations. Hence, we quantified relationships between the Omega-3 Index and measures of adiposity in the present study, even though it was evident that erythrocyte DHA alone was a stronger predictor of adiposity than the combination of EPA + DHA.

Whilst there was no apparent relationship between the Omega-3 Index and measures of adiposity in men, examination of quartiles of the Omega-3 Index in women revealed a non-linear relationship with BMI (Figure 1), wherein BMI was similar in the two highest quartiles but rose sharply in the lower quartiles. This was even more evident when curvilinear relationships were tested. The best fit (shown in Figure 2) was a sigmoidal curve, indicating a plateau effect within an approximate range of 5%–9%, below which BMI appeared to increase exponentially. Bearing in mind that the Omega-3 Index predicts greater risk of cardiovascular disease below 4% and lesser risk above 8%, it appears that extremes of the Omega-3 Index may also be associated with other risk factors, including adiposity and depression [24,25].

BMI is a relatively crude measure of obesity; gender differences may reflect differences between men and women in the relative contribution of fat and lean mass to BMI. However, significant curvilinear relationships were found in women between the Omega-3 Index and both % body fat and waist circumference as well as BMI, strengthening the argument that omega-3 intake is inversely related to adiposity in women.

In conclusion, the outcomes of this cross-sectional analysis of erythrocyte LC *n*-3 PUFA content and measures of adiposity in a convenience sample of Australian adults are consistent with other evidence suggesting an inverse relationship between LC *n*-3 PUFA intakes and obesity. In particular, DHA intake was a negative predictor of BMI, waist circumference and body fat content in women, whereas DPA was a weaker negative predictor of body fat content in men. Analysis of the Omega-3 Index indicates that women in the lower range of the Index may have increased risk of obesity. These data warrant further confirmation in larger studies where potential gender-specific effects of individual LC *n*-3 PUFA are also taken into account.

## Abbreviations

BMI: body mass index
WC: waist circumference
BF: body fat
LC *n*-3 PUFA: long-chain omega-3 polyunsaturated fatty acids
EPA: eicosapentaenoic acid
DPA: docosapentaenoic acid
DHA: docosahexaenoic acid
